# Identification and Validation of Inflammatory Response-Related Gene Signatures to Predict the Prognosis of Neuroblastoma

**DOI:** 10.1155/2022/2417351

**Published:** 2022-04-30

**Authors:** Jiye Song, Liang Song, Zhenmei Lv, Jianke Liu, Xuan Feng, Song Zhang, Aiqin Song

**Affiliations:** ^1^Department of Pediatric Medical Center, The Affiliated Hospital of Qingdao University, Qingdao, China; ^2^Qingdao Medical College, Qingdao University, Qingdao, China; ^3^Qingdao Women and Children's Hospital, Qingdao University, China

## Abstract

**Background:**

Neuroblastoma (NB) is the third most common malignant tumor in children. The inflammation is believed to be closely related to NB patients' prognosis. However, there is no comprehensive research to study the role of inflammatory response-related gene (IRRG) in NB patients.

**Methods:**

We downloaded the gene expression profiles of NB patients from GEO and TARGET database, and the expression of 200 IRRGs was extracted. Then, we performed differentially analysis between INSS stage 4 and INSS stage 4S NB patients. The univariate and multivariate Cox regression analyses were performed to screen out the overall survival- (OS-) and event-free survival- (EFS-) related IRRGs in GSE49710, and two signatures were constructed; both signatures were evaluated by Kaplan-Meier (K-M) survival curve and receiver operating characteristic (ROC) curve. Finally, the TARGET cohort was used to validate IRRG signatures, and the independence of the prognostic IRRG signatures was evaluated by integrating clinical information.

**Results:**

We screened out 10 OS-related IRRGs and 11 EFS-related IRRGs. Then, we identified that OS- and EFS-related IRRG signatures and found that the OS and EFS of NB patients in the low-risk group were significantly superior than those in the high-risk group (both *P* value < 0.0001). The AUC values of 3-, 5-, and 7-year OS are 0.910, 0.933, and 0.921, respectively, and 3-, 5-, and 7-year EFS are 0.840, 0.835, and 0.837, respectively. In addition, we found that both IRRG signatures can be used as independent prognostic indicators for patients with NB. Both IRRG signatures still have good predictive ability in validation cohort.

**Conclusions:**

We constructed and validated two prognostic gene signatures based on IRRGs. Our study helped us to better understand the role of inflammation in NB and provided new insights for the prognosis assessment and treatment strategy for NB patients.

## 1. Background

Neuroblastoma (NB) is a common substantial malignant tumor in children, and the incidence of NB is second only to acute lymphoblastic leukemia (ALL) and brain and central nervous system (CNS) tumors, accounting for 7% of the cancer occurrence among children and adolescents [1]. The current standard treatment methods for NB patients include chemotherapy, surgery, radiotherapy, immunotherapy, and stem cell transplantation after high-dose chemotherapy [2–4]. With the improvement of the treatment options, the overall survival (OS) rate of NB patients has been significantly increased from 54% in 1975-1979 to 79% in 2003-2009 [1]. Unfortunately, for high-risk NB patients, their 5-year OS is only 40-50% [5–7], and 5-year event-free survival (EFS) is less than 50% [8]. At present, studies have proved that age and stage tumor genomics are crucial to the risk stratification of NB patients [9], but this is far from enough. It is still necessary to explore and find out the prognostic biomarkers to further improve the treatment strategies and the survival of NB patients.

Inflammation is closely related to the tumorigenesis and progression. Persistent chronic inflammation can often promote the growth and invasion of malignant cells, promote angiogenesis, and even affect the therapeutic effect [10–13]. For example, Yuan et al. have found that the high expression levels of inflammatory, such as C-reactive protein, interleukin-6, and tumor necrosis factor-*α* receptor 2 biomarkers and a long-term proinflammatory dietary pattern, were associated with poorer survival in pancreatic cancer patients [14, 15]. Except for the study of transcriptomics, Candido et al. concluded that inflammatory epigenetic markers have predictive value on the clinical outcome and survival of cancer patients [16]. And the CheckMate 040 trial (NCT01658878) found that multiple inflammatory biomarkers and inflammatory signature were significantly associated with the survival of hepatocellular carcinoma (HCC) patients and influenced the efficacy of nivolumab treatment and had a certain predictive value [17]. What is more, several bioinformatics-based studies confirmed that IRRG-based signature is a robust prognostic biomarker for cancer patients [18–22]. Lin et al. constructed eight IRRG signatures in HCC patients and found that it cannot only predict the prognosis of patients but also affect tumor microenvironment, immune response, and drug sensitivity to a certain extent [20]. In addition, other studies have found that the use of anti-inflammatory drugs, such as aspirin, can effectively reduce the incidence rate and risk of distant metastasis of cancers, such as colorectal cancer, esophageal cancer, gastric cancer, biliary tract cancer, and breast cancer [23–25]. Due to the importance of inflammation in cancer patients' prognosis, it is necessary to construct an effective IRRG signature to predict the survival of NB patients.

The prognosis of NB patients in INSS stages 4 and 4S is significantly different; that is, compared with patients with INSS stage 4, patients with INSS stage 4S are usually of low risk and have superior outcomes with 5-year OS rates was 91% ± 1% [6]. In addition, there have been many studies on the differences between INSS stage 4 and 4S patients [26–28]. Therefore, in our study, we compared the inflammatory response-related genes (IRRGs) of NB patients between INSS stage 4 and 4S in Gene Expression Omnibus (GEO) database to obtain differentially expressed IRRGs and further analyzed them to obtain prognostic IRRGs to construct gene signatures. Finally, the accuracy of the signature prediction was verified in Therapeutically Applicable Research To Generate Effective Treatments (TARGET) database. These findings help us to better understand the relationship between inflammation and prognosis of NB and provide us new insights into treatment targets and prognostic indicators for patients with NB.

## 2. Methods

### 2.1. Data Collection and Preprocessing

**Figure 1:**
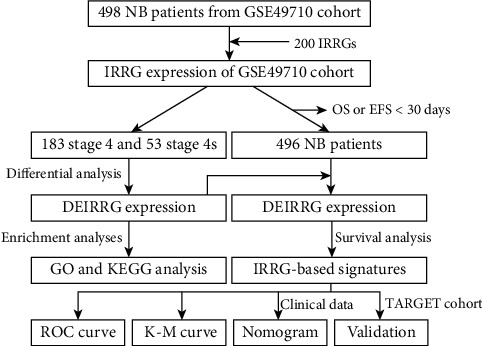


We downloaded the gene expression data and clinical information of 498 histologically diagnosed NB patients (GSE49710) from GEO database to form as the training cohort. Patients with OS or EFS less than 30 days were excluded, and 496 histologically diagnosed NB patients were included. Meanwhile, based on the same criteria of GEO cohort, a total of 153 NB patients from TARGET database were used as the validation cohort. For GEO dataset, the normalized matrix files were directly downloaded, and for TARGET dataset, the RNA sequencing data (FPKM value) of gene expression were downloaded and transformed into transcripts per kilobase million (TPM) values [29]. Then, the “ComBat” algorithm of sva package was performed to correct the batch effects from nonbiological technical biases [29]. The flowchart of our research was shown in Figure 1.

### 2.2. Identification of Differentially Expressed IRRGs and Enrichment Analyses

The list of inflammatory response-related genes (IRRGs) was downloaded from the Molecular Signatures database (Supplementary Table 1) [20]. To obtain differentially expressed IRRGs, we compared the expression of IRRGs between NB patients with INSS stage 4 and INSS stage 4S in GSE49710 dataset. Genes with false discovery rate (FDR) < 0.05 were defined as differentially expressed IRRGs (DEIRRGs), and the results of differential analysis were visualized by volcano plot and heat map. To further explore the potential function of identified DEIRRGs, we performed enrichment analyses, including Gene Ontology (GO) functional annotation and Kyoto Encyclopedia of Genes and Genomes (KEGG) pathway enrichment analyses. The enrichment analyses were performed in the Metascape database [30].

### 2.3. Identification and Analysis of the IRRG-Based Prognostic Signatures

In order to make the results of survival analysis more accurate, we excluded patients whose OS or EFS less than 30 days, and 496 patients in GSE49710 dataset were selected for the survival analyses. Then, we performed univariate Cox regression analyses to determine the OS- and EFS-related IRRGs, respectively. Next, the LASSO analysis and multivariate Cox regression analysis were adopted to avoid overfitting and ultimately to screen out the significant OS- and EFS-related IRRGs. Finally, two prognostic signatures were constructed for predicting the OS and EFS by liner combining the expression values of the IRRGs weighted with its regression coefficients of the multivariate Cox regression analysis, and the specific formula is as follows:

(1) $$\begin{array}{c} \text{risk score}=\sum \left(\beta_{n}\times \text{expression of }{\text{gene}}_{n}\right). \end{array}$$

Each patient got their own risk score according to the formula, and they were divided into high-risk group and low-risk group according to the median risk score. Then, Kaplan-Meier (K-M) survival curves and the log-rank test were performed to compare the prognostic difference between these two groups. The time-dependent receiver operating characteristic (ROC) curves and area under the curve (AUC) value were adopted to evaluate the predictive power of the IRRG-based signatures.

### 2.4. Evaluating the Independence of Signatures and Developing Nomogram

Furthermore, to verify whether the IRRG signature can be used as a reliable independent prognostic indicator, we combined the risk score model with the clinical characteristics including age, sex, MYCN status, and INSS stage of neuroblastoma patients and conducted the univariate and multivariate Cox regression analysis to analyze the prognostic impact. Then, based on the independent prognostic factors, two prognostic nomograms were developed by the “rms” package in the R software for predicting OS and EFS of NB patients. The concordance index (C-index) and calibration curve were generated to assess the discrimination and calibration of the nomograms.

### 2.5. External Validation of IRRG-Based Prognostic Signatures

External validation is vital for prognostic signature. Therefore, we further validate two signatures in the independent cohort. In the TARGET cohort, a total of 153 patients with OS or EFS longer than 30 days were included. The risk scores of 153 patients were calculated according to the formula constructed in the training cohort. Then, according to the cut-off value identified in the training cohort, all patients were divided into high- and low-risk groups. ROC curves, AUC values, and K-M survival curves were conducted to validate the predictive ability of IRRG signatures.

### 2.6. Statistical Analyses

All statistical analyses in this study were performed in R 3.6.1. A *P* value < 0.05 (two-tailed) was considered to indicate statistical significance. Univariate Cox analysis, LASSO analysis, and multivariate Cox regression analysis were selected for survival analyses and construct prognostic signature. K-M survival curves and the log-rank test were performed to compare the prognostic difference between different risk groups. ROC curves and AUC value were used to evaluate the discrimination of the IRRG-based signatures.

## 3. Results

### 3.1. Screening of Differentially Expressed Inflammatory Response-Related Genes

**Figure 2:**
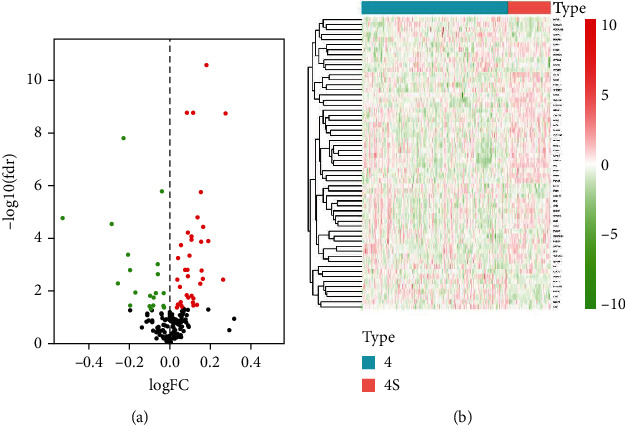


**Figure 3:**
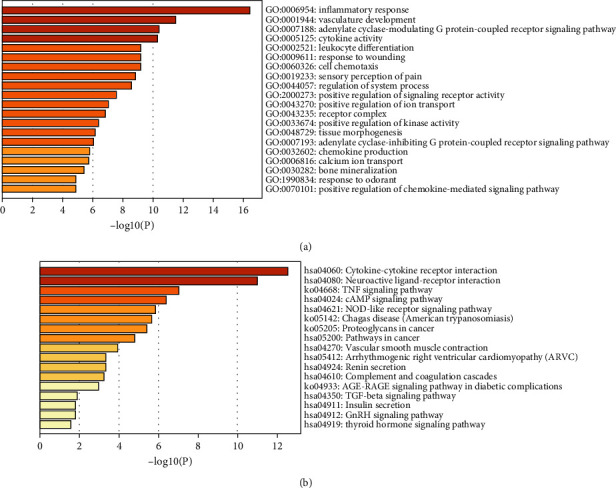


By integrating the gene expression profiles of NB patients with the IRRGs downloaded from the Molecular Signatures database, a profile with 193 IRRG expressions from NB patients both in GEO dataset and TARGET dataset was obtained. Then, by comparing the 193 IRRGs between 183 INSS stage 4 and 53 INSS stage 4S NB patients in the GSE49710 cohort, we extracted 58 DEIRRGs, which were visualized in the volcano plot and heat map (Figures 2(a) and 2(b)). In the GO functional analyses, the results indicated that DEIRRGs were mainly involved in inflammatory response, vasculature development, leukocyte differentiation, cell chemotaxis, chemokine production, and positive regulation of chemokine-mediated signaling pathway (Figure 3(a)). KEGG analysis suggested that DEIRRGs were mainly associated with cytokine-cytokine receptor interaction, TNF signaling pathway, NOD-like receptor signaling pathway, pathways in cancer, and TGF-beta signaling pathway (Figure 3(b)). Generally, the enrichment analyses showed that DEIRRGs not only play an important role in the inflammatory response but are also associated with tumorigenesis and tumor immune microenvironment.

### 3.2. Identification of Prognostic DEIRRGs for NB Patients

**Figure 4:**
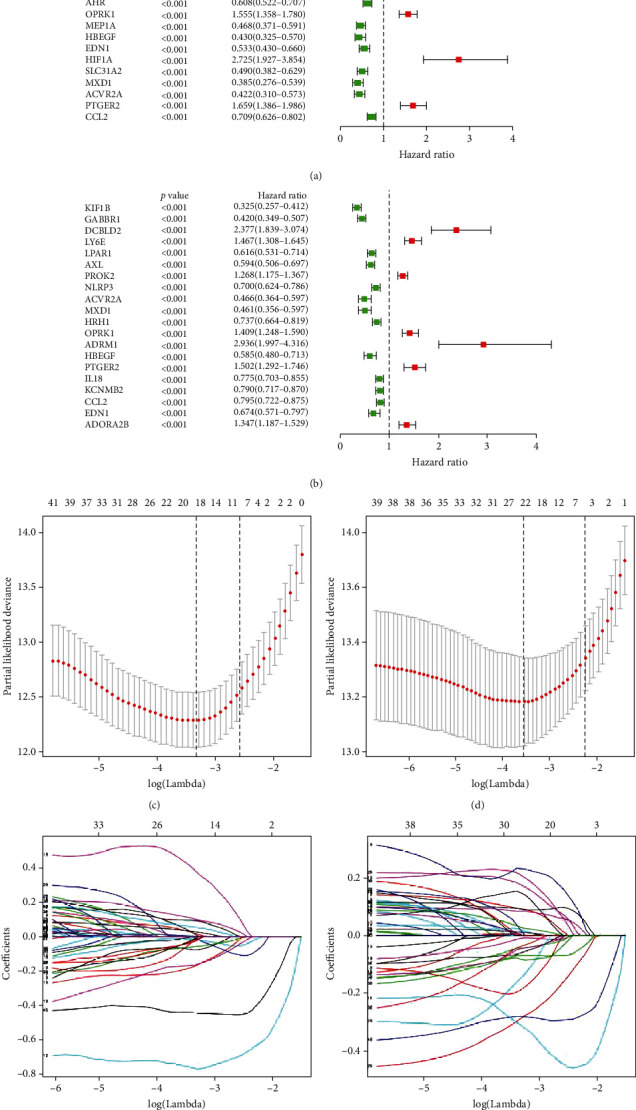


**Figure 5:**
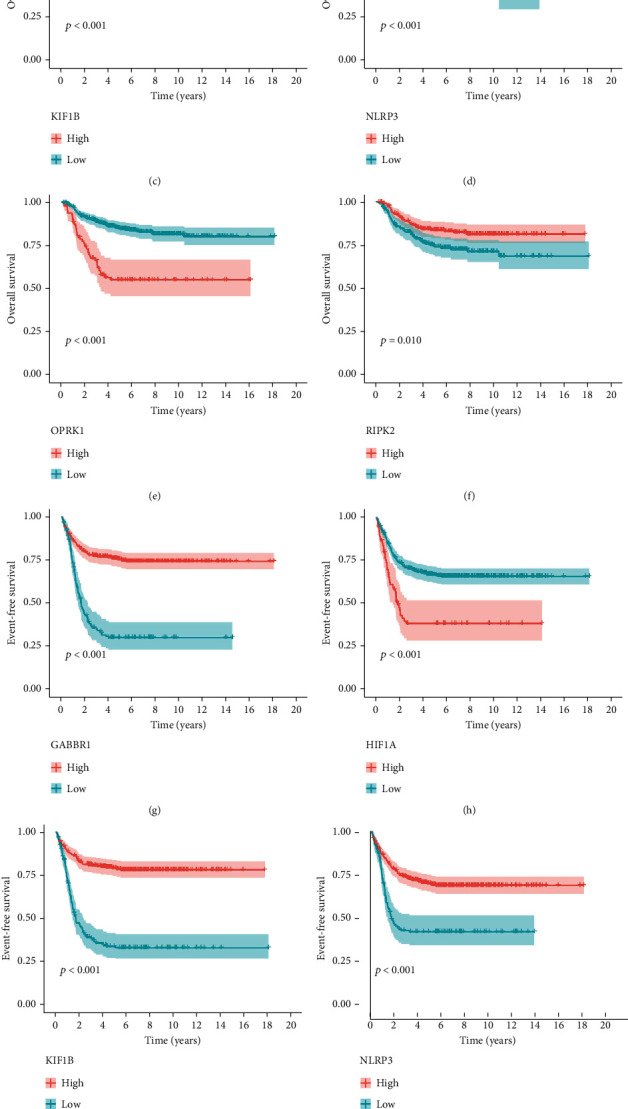


Based on the DEIRRG, we preliminarily screened out 45 OS-related IRRGs and 43 EFS-related DEIRRGs by univariate Cox regression analysis and displayed their top 20 significant DEIRRGs by forest map (Figures 4(a) and 4(b)). Next, by performing the LASSO (Figures 4(c)–4(f)) analysis and multivariate Cox regression analysis, we ultimately identified significant OS- and EFS-related DEIRRGs and obtained their respective correlation coefficients, including 10 OS-related DEIRRGs (Table 1) and 11 EFS-related DEIRRGs (Table 2). Among these DEIRRGs, genes with an HR < 1 indicate that they are protective factors, which is conducive to the prognosis of patients, while genes with an HR > 1 indicate the opposite. Among them, a total of six DEIRRGs showed to be associated with both OS and EFS. The K-M survival curves indicated that there was a significant difference in prognosis between the low- and high-expression groups (Figure 5).

### 3.3. Constructing Two IRRG-Based Prognostic Signatures

**Figure 6:**
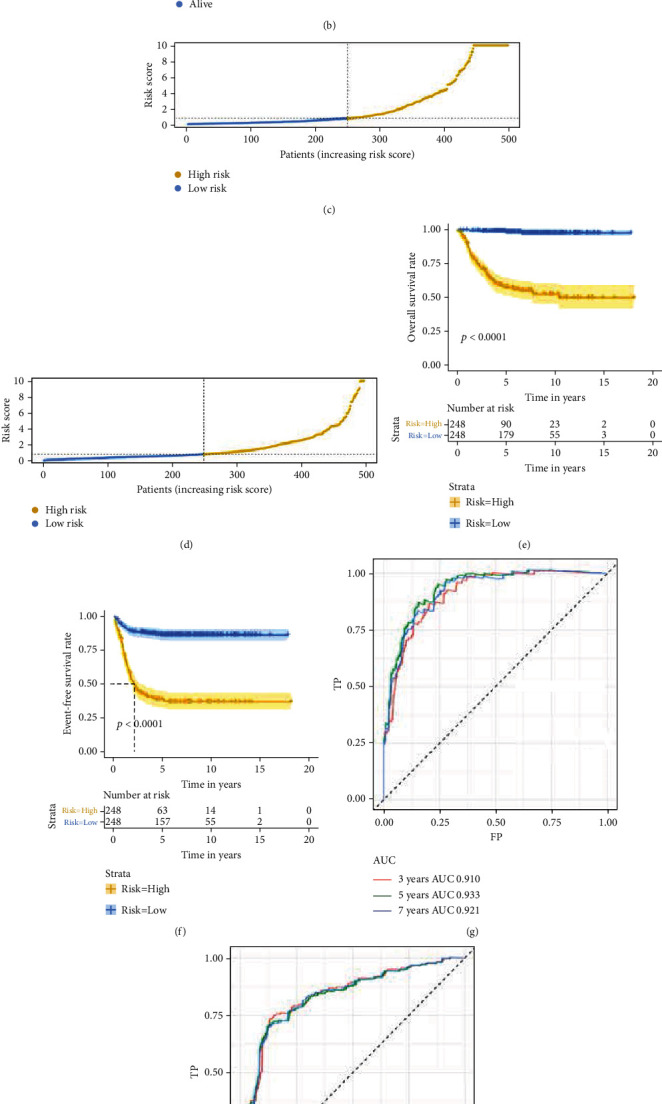


According to the methods, two prognostic signatures based on OS- and EFS-related IRRGs were established, and risk score of each patient was calculated (Figures 6(a) and 6(b)). According to the median risk score, we divided the patients into low- and high-risk groups (Figures 6(c) and 6(d)). The K-M survival analysis showed that the OS and EFS of NB patients in the low-risk group were significantly superior than those in the high-risk group (*P* value < 0.0001) (Figures 6(e) and 6(f)). In addition, ROC curve analysis indicated that the AUC values of OS signature in 3-, 5-, and 7-year were 0.910, 0.933, and 0.921, respectively (Figure 6(g)), and the AUC values of EFS signature in 3-, 5-, and 7-year were 0.840, 0.835, and 0.837, respectively (Figure 6(h)), indicating that our prognosis signatures have favorable discrimination.

### 3.4. IRRG-Based Signatures Were Independent Prognostic Indicators for NB Patients

By performing the univariate Cox regression analysis for IRRG-based signatures and clinicopathological features, we found that age, MYCN status, INSS stage, and IRRG-based signatures were remarkably correlated with both OS and EFS except sex (Table 3). Then, the multivariate Cox regression analysis was carried out, and the results showed that age, MYCN status, INSS stage, and IRRG-based signature were still significantly related to the OS of NB patients (Table 4). However, only age, INSS stage, and IRRG-based signature were markedly associated with EFS of NB patients (Table 4). Generally, according to the above results, we can know that the IRRG-based signatures can independently predict the prognosis of patients with NB, which further proved the reliability of our study.

### 3.5. Development of Nomograms Based on IRRG-Based Signatures and Clinical Parameters

**Figure 7:**
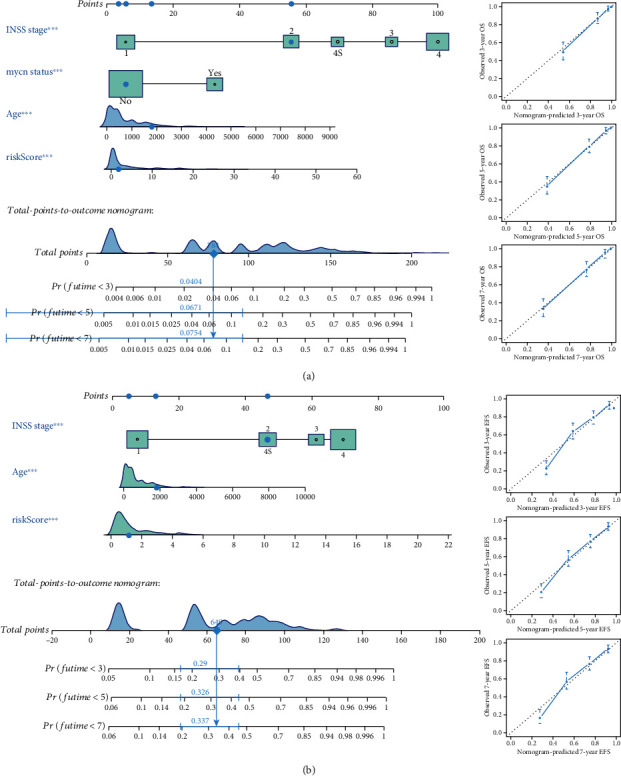


On the basis of independent prognostic clinical parameters, two nomograms were developed to predict the OS and EFS of NB patients, respectively (Figures 7(a) and 7(b)). The values of C-index of OS and EFS nomograms were 0.873 and 0.754, respectively. The calibration curve suggested that the nomogram-predicted prognosis was highly consistent with the observed prognosis, which means the favorable calibration of both nomograms (Figures 7(a) and 7(b)).

### 3.6. Validating the Prognostic Risk Score Models in TARGET Cohort

**Figure 8:**
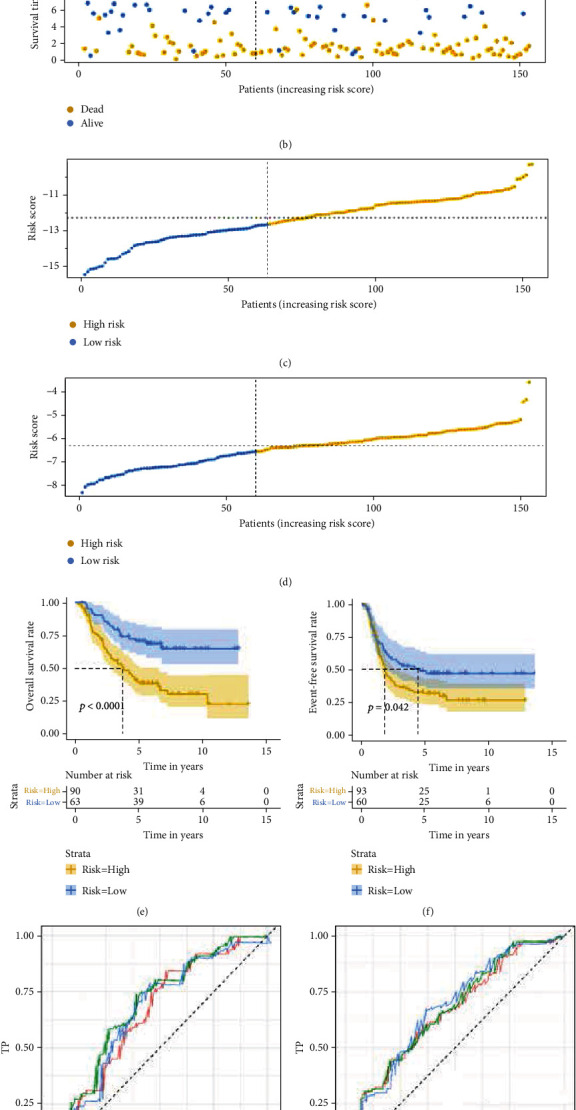


We verified the constructed two IRRG-based signatures in the TARGET cohort. Firstly, we calculated the risk score of TARGET cohort and divided them into high- and low-risk groups according to the same cut-off values identified in the training cohort (Figures 8(a)–8(d)). K-M survival curves showed that the OS and EFS of the high-risk group were significantly worse than those in the low-risk group (Figures 8(e) and 8(f)). The AUC values of 3-, 5-, and 7-year OS are 0.660, 0.692, and 0.664, respectively (Figure 8(g)), and 3-, 5-, and 7-year EFS are 0.660, 0.667, and 0.674, respectively (Figure 8(h)).

## 4. Discussion

Inflammation is closely related to the occurrence and development of tumor, and there are various related studies that have been carried out in NB [31]. For example, Zheng et al. found that patients with higher C-reactive protein to albumin ratio (CAR) and elevated high-sensitivity modified Glasgow Prognostic Score (Hs-mGPS) had significantly worse by analyzing blood inflammatory biomarkers or inflammation based scores in patients with NB [32]. Asgharzadeh et al. found that tumor-associated inflammatory cells were also significantly associated with prognosis in patients with NB, especially tumor-associated macrophages (TAMs), which were more infiltrated in metastatic NB than localized NB, and by integrating tumor-related genes and inflammation-related genes, they constructed a 14-gene signature to predict NB patients (age ≥ 18 months) and diagnosed with metastatic MYCN-nonamplified (NBL-NA), which provided a significant prognostic marker for patients with this type [33]. In addition, several studies confirmed that the high expression of the inflammatory factor IL-6 was associated with the disease progression in patients with NB and helped the cancer cells to escape the killing of drugs, which was one of the indicators of poor prognosis [34, 35]. In this study, two gene signatures were constructed from the perspective of IRRGs to predict the prognosis of NB patients, and a good predictive effect was achieved.

In our research, we took an intersection of the genes of NB patients and IRRGs and obtained the expression of 193 IRRGs of NB patients. Next, we compared these genes between 183 INSS stage 4 and 53 INSS stage 4S NB patients in GSE49710 dataset, and 58 DEIRRGs were identified. Then, by performing survival analysis, we identified 10 OS-related DEIRRGs and 11 EFS-related DEIRRGs. Based on these prognostic DEIRRGs, we constructed two signatures and found that NB patients in the low-risk group had superior outcomes than high-risk patients. What is more, we verified the independence of the prognostic signatures with clinicopathological features and further developed two prognostic nomograms to predict the prognosis of patients with NB. Finally, we verified the accuracy of the IRRG-based signatures in the external validation cohort and confirmed favorable stability.

Among these prognostic DEIRRGs included in the final signatures, there were six DEIRRGs that were associated with both OS and EFS (OPRK1, RIPK2, NLRP3, KIF1B, HIF1A, and GABBR1). All of them were closely associated with tumorigenesis and progression. Opioid receptor kappa-1 (OPRK1) is one of the G-protein-coupled receptors, involved in G protein-coupled receptor signaling pathway, pain perception, synaptic transmission, and other processes [36]. It has been reported that it was highly expressed in small bowel neuroendocrine tumors and pancreatic neuroendocrine tumors and can predict primary tumors according to the expression of OPRK1 in metastatic tumors, which is expected to become a new promising therapeutic target [37, 38]. Receptor-interacting serine/threonine-protein kinase 2 (RIPK2) plays a vital role in the regulation of innate and adaptive immune responses [39, 40], and its high expression has been found to be closely associated with poor prognosis of human kidney renal clear cell carcinoma [41], gastric cancer [42], inflammatory breast cancer (IBC) [43], glioblastoma [44, 45], and so on, and is a potential therapeutic target for a variety of cancers. NACHT, LRR, and PYD domain-containing protein 3 (NLRP3) plays an essential role in innate immunity, inflammation, and metabolize [46–48]. Some studies have found that NLRP3 is a potential protective factor, such as the lack of NLRP3 inflammasome will increase the metastatic growth of colorectal cancer in the liver [49] and the activation of NLRP3 inflammasome may suppress the growth of tumor cells [50]. Kinesin family member 1B (KIF1B) is a tumor suppressor gene, and many studies have found that KIF1B*β* can induce cell apoptosis in NB, and NB patients with poor prognosis are usually accompanied by deletion of chromosome 1p36, where KIF1B was located [51–54]. Hypoxia-inducible factor 1-alpha (HIF1A) is the main transcriptional regulator of the adaptive response to hypoxia, which is related to metastasis of various tumor cells and poor prognosis of cancer patients [55, 56], including sarcoma [57, 58], advanced renal cell carcinoma (RCC) [59], breast cancer [60], and NB [61–63]. Gamma-aminobutyric acid type B receptor subunit 1 (GABBR1) is a neurotransmitter receptor [64]; it has been reported that it can improve the proliferation of hematopoietic stem/progenitor cells (HSPC) which showed potential benefits for clinical transplantation [65]. However, there are few studies on the relationship between GABBR1 and cancer, and further exploration is needed. Overall, the role of these genes in previous studies is similar to our results, which makes our findings more reliable.

There is no denying that this research is not perfect. All of our results were obtained by analyzing the patient information and gene expression profiles in public databases, and no clinical specimens were collected for further verification. Moreover, the specific mechanism of each gene in this study is still unclear, and it is an important research direction in the future. However, we have validated our risk score models in an independent database and obtained good results, which to some extent makes up for our limitations.

## 5. Conclusion

In summary, two robust IRRG-based prognostic signatures were constructed to predict NB patients' OS and EFS, respectively. Furthermore, by integrating clinical information, we constructed that two nomograms further validated the ability of the risk score models as independent predictive indicators. Collectively, this study provides new insights for the prognosis assessment and treatment strategy of NB patients and further experimental. The specific mechanisms of action of these genes will need to be further identified in the future.

## Figures and Tables

**Table 1:** The information of 10 OS-related IRRGs by multivariate Cox regression analysis.

Gene	Coef	HR	95% CI	*P* value
OPRK1	0.188	1.21	1.00-1.45	0.042
RIPK2	-0.514	0.60	0.39-0.91	0.017
NLRP3	-0.166	0.85	0.72-1.00	0.051
KIF1B	-0.926	0.40	0.26-0.61	<0.001
HIF1A	0.765	2.15	1.44-3.20	<0.001
MEP1A	-0.317	0.73	0.57-0.93	0.011
HRH1	-0.226	0.80	0.68-0.94	0.007
BEST1	0.185	1.20	0.94-1.54	0.145
LY6E	0.137	1.15	0.95-1.38	0.146
GABBR1	-0.568	0.57	0.41-0.78	<0.001

OS: overall survival; IRRG: inflammatory response-related gene; HR: hazard ratio; CI: confidence interval.

**Table 2:** The information of 11 EFS-related IRRGs by multivariate Cox regression analysis.

Gene	Coef	HR	95% CI	*P* value
GNAI3	0.406	1.50	1.04-2.17	0.031
PTGER2	0.158	1.17	1.00-1.38	0.057
OPRK1	0.153	1.17	1.00-1.35	0.044
RIPK2	-0.470	0.62	0.47-0.83	0.001
NLRP3	-0.139	0.87	0.77-0.99	0.028
KIF1B	-0.422	0.66	0.47-0.91	0.013
HIF1A	0.309	1.36	1.00-1.84	0.046
ACVR2A	-0.283	0.75	0.54-1.05	0.097
ATP2B1	0.318	1.37	1.05-1.80	0.021
LPAR1	-0.379	0.68	0.57-0.82	<0.001
GABBR1	-0.274	0.76	0.60-0.97	0.024

EFS: event-free survival; IRRG: inflammatory response-related gene; HR: hazard ratio; CI: confidence interval.

**Table 3:** Univariate Cox regression analyses for identifying prognostic clinical characteristics.

	Univariate analysis (OS)			Univariate analysis (EFS)		
	HR	95% CI	*P* value	HR	95% CI	*P* value
Risk score	1.08	1.07-1.10	<0.001	1.23	1.20-1.28	<0.001
Sex	0.81	0.55-1.19	0.288	0.90	0.67-1.21	0.492
Age	1.0003	1.0002-1.0004	<0.001	1.0003	1.0002-1.0004	<0.001
MYCN status	7.67	5.17-11.27	<0.001	3.20	2.34-4.40	<0.001
INSS stage						
Stage 1						
Stage 2	6.39	0.71-57.17	0.097	3.73	1.69-8.23	0.001
Stage 3	30.20	3.97-229.66	<0.001	7.63	3.59-16.24	<0.001
Stage 4	78.58	10.93-564.99	<0.001	12.49	6.33-24.66	<0.001
Stage 4S	9.63	1.07-86.19	0.043	3.42	1.44-8.11	0.005

OS: overall survival; EFS: event-free survival; HR: hazard ratio; CI: confidence interval.

**Table 4:** Multivariate Cox regression analyses for identifying prognostic clinical characteristics.

	Multivariate analysis (OS)			Multivariate analysis (EFS)		
	HR	95% CI	*P* value	HR	95% CI	*P* value
Risk score	1.05	1.03-1.07	<0.001	1.16	1.11-1.21	<0.001
Age	1.0003	1.0002-1.0004	<0.001	1.0002	1.0001-1.0003	<0.001
MYCN status	2.84	1.87-4.31	<0.001	1.26	0.87-1.81	0.216
INSS stage						
Stage 1						
Stage 2	6.99	0.78-62.78	0.082	3.76	1.70-8.31	0.001
Stage 3	22.66	2.96-173.40	0.003	5.80	2.70-12.46	<0.001
Stage 4	39.19	5.40-284.22	<0.001	7.66	3.82-15.37	<0.001
Stage 4S	12.02	1.33-108.68	0.027	3.90	1.63-9.32	0.002

OS: overall survival; EFS: event-free survival; HR: hazard ratio; CI: confidence interval.

## Data Availability

The datasets presented in this study can be found in online repositories. The names of the repository/repositories and accession number(s) can be found in the article/supplementary material.

## Supplementary Materials

Supplementary Materials Table S1: the list of inflammatory response-related genes.
