# Supplementary material for: Identification and Validation of Inflammatory Response-Related Gene Signatures to Predict the Prognosis of Neuroblastoma
Source: Int J Genomics. 2022 Apr 30;2022:2417351. doi: 10.1155/2022/2417351 (PMC9078807; doi:10.1155/2022/2417351)
Supplement: Supplementary Materials — Table S1: the list of inflammatory response-related genes. [file 2417351.f1.pdf]

GeneSymbol	GeneDescription
ABCA1	ATP binding cassette subfamily A member 1
ABI1	abl interactor 1
ACVR1B	activin A receptor type 1B
ACVR2A	activin A receptor type 2A
ADM	adrenomedullin
ADORA2B	adenosine A2b receptor
ADRM1	adhesion regulating molecule 1
AHR	aryl hydrocarbon receptor
APLNR	apelin receptor
AQP9	aquaporin 9
ATP2A2	ATPase sarcoplasmic/endoplasmic reticulum Ca2+ transporting 2
ATP2B1	ATPase plasma membrane Ca2+ transporting 1
ATP2C1	ATPase secretory pathway Ca2+ transporting 1
AXL	AXL receptor tyrosine kinase
BDKRB1	bradykinin receptor B1
BEST1	bestrophin 1
BST2	bone marrow stromal cell antigen 2
BTG2	BTG anti-proliferation factor 2
C3AR1	complement C3a receptor 1
C5AR1	complement C5a receptor 1
CALCRL	calcitonin receptor like receptor
CCL17	C-C motif chemokine ligand 17
CCL2	C-C motif chemokine ligand 2
CCL20	C-C motif chemokine ligand 20
CCL22	C-C motif chemokine ligand 22
CCL24	C-C motif chemokine ligand 24
CCL5	C-C motif chemokine ligand 5
CCL7	C-C motif chemokine ligand 7
CCR7	C-C motif chemokine receptor 7
CCRL2	C-C motif chemokine receptor like 2
CD14	CD14 molecule
CD40	CD40 molecule
CD48	CD48 molecule
CD55	CD55 molecule (Cromer blood group)
CD69	CD69 molecule
CD70	CD70 molecule
CD82	CD82 molecule
CDKN1A	cyclin dependent kinase inhibitor 1A
CHST2	carbohydrate sulfotransferase 2
CLEC5A	C-type lectin domain containing 5A
CMKLR1	chemerin chemokine-like receptor 1
CSF1	colony stimulating factor 1
CSF3	colony stimulating factor 3
CSF3R	colony stimulating factor 3 receptor
CX3CL1	C-X3-C motif chemokine ligand 1
CXCL10	C-X-C motif chemokine ligand 10
CXCL11	C-X-C motif chemokine ligand 11
CXCL6	C-X-C motif chemokine ligand 6
CXCL9	C-X-C motif chemokine ligand 9
CXCR6	C-X-C motif chemokine receptor 6
CYBB	cytochrome b-245 beta chain
DCBLD2	discoidin, CUB and LCCL domain containing 2
EBI3	Epstein-Barr virus induced 3

EDN1	endothelin 1
EIF2AK2	eukaryotic translation initiation factor 2 alpha kinase 2
EMP3	epithelial membrane protein 3
ADGRE1	adhesion G protein-coupled receptor E1
EREG	epiregulin
F3	coagulation factor III, tissue factor
FFAR2	free fatty acid receptor 2
FPR1	formyl peptide receptor 1
FZD5	frizzled class receptor 5
GABBR1	gamma-aminobutyric acid type B receptor subunit 1
GCH1	GTP cyclohydrolase 1
GNA15	G protein subunit alpha 15
GNAI3	G protein subunit alpha i3
GP1BA	glycoprotein Ib platelet subunit alpha
GPC3	glypican 3
GPR132	G protein-coupled receptor 132
GPR183	G protein-coupled receptor 183
HAS2	hyaluronan synthase 2
HBEGF	heparin binding EGF like growth factor
HIF1A	hypoxia inducible factor 1 subunit alpha
HPN	hepsin
HRH1	histamine receptor H1
ICAM1	intercellular adhesion molecule 1
ICAM4	intercellular adhesion molecule 4
ICOSLG	inducible T cell costimulator ligand
IFITM1	interferon induced transmembrane protein...
IFNAR1	interferon alpha and beta receptor subun...
IFNGR2	interferon gamma receptor 2
IL10	interleukin 10
IL10RA	interleukin 10 receptor subunit alpha
IL12B	interleukin 12B
IL15	interleukin 15
IL15RA	interleukin 15 receptor subunit alpha
IL18	interleukin 18
IL18R1	interleukin 18 receptor 1
IL18RAP	interleukin 18 receptor accessory protein
IL1A	interleukin 1 alpha
IL1B	interleukin 1 beta
IL1R1	interleukin 1 receptor type 1
IL2RB	interleukin 2 receptor subunit beta
IL4R	interleukin 4 receptor
IL6	interleukin 6
IL7R	interleukin 7 receptor
CXCL8	C-X-C motif chemokine ligand 8
INHBA	inhibin subunit beta A
IRAK2	interleukin 1 receptor associated kinase 2
IRF1	interferon regulatory factor 1
IRF7	interferon regulatory factor 7
ITGA5	integrin subunit alpha 5
ITGB3	integrin subunit beta 3
ITGB8	integrin subunit beta 8
KCNA3	potassium voltage-gated channel subfamily A member 3
KCNJ2	potassium inwardly rectifying channel subfamily J member 2
KCNMB2	potassium calcium-activated channel subfamily M regulatory beta subunit 2

KIF1B	kinesin family member 1B
KLF6	Kruppel like factor 6
LAMP3	lysosomal associated membrane protein 3
LCK	LCK proto-oncogene, Src family tyrosine kinase
LCP2	lymphocyte cytosolic protein 2
LDLR	low density lipoprotein receptor
LIF	LIF interleukin 6 family cytokine
LPAR1	lysophosphatidic acid receptor 1
LTA	lymphotoxin alpha
LY6E	lymphocyte antigen 6 family member E
LYN	LYN proto-oncogene, Src family tyrosine kinase
MARCO	macrophage receptor with collagenous structure
MEFV	MEFV innate immuity regulator, pyrin
MEP1A	meprin A subunit alpha
MET	MET proto-oncogene, receptor tyrosine kinase
MMP14	matrix metallopeptidase 14
MSR1	macrophage scavenger receptor 1
MXD1	MAX dimerization protein 1
MYC	MYC proto-oncogene, bHLH transcription factor
NAMPT	nicotinamide phosphoribosyltransferase
NDP	norrin cystine knot growth factor NDP
NFKB1	nuclear factor kappa B subunit 1
NFKBIA	NFKB inhibitor alpha
NLRP3	NLR family pyrin domain containing 3
NMI	N-myc and STAT interactor
NMUR1	neuromedin U receptor 1
NOD2	nucleotide binding oligomerization domain containing 2
NPFFR2	neuropeptide FF receptor 2
OLR1	oxidized low density lipoprotein receptor 1
OPRK1	opioid receptor kappa 1
OSM	oncostatin M
OSMR	oncostatin M receptor
P2RX4	purinergic receptor P2X 4
P2RX7	purinergic receptor P2X 7
P2RY2	purinergic receptor P2Y2
PCDH7	protocadherin 7
PDE4B	phosphodiesterase 4B
PDPN	podoplanin
PIK3R5	phosphoinositide-3-kinase regulatory subunit 5
PLAUR	plasminogen activator, urokinase receptor
PROK2	prokineticin 2
PSEN1	presenilin 1
PTAFR	platelet activating factor receptor
PTGER2	prostaglandin E receptor 2
PTGER4	prostaglandin E receptor 4
PTGIR	prostaglandin I2 receptor
PTPRE	protein tyrosine phosphatase receptor type E
PVR	PVR cell adhesion molecule
RAF1	Raf-1 proto-oncogene, serine/threonine kinase
RASGRP1	RAS guanyl releasing protein 1
RELA	RELA proto-oncogene, NF-kB subunit
RGS1	regulator of G protein signaling 1
RGS16	regulator of G protein signaling 16
RHOG	ras homolog family member G

RIPK2	receptor interacting serine/threonine kinase 2
RNF144B	ring finger protein 144B
ROS1	ROS proto-oncogene 1, receptor tyrosine kinase
RTP4	receptor transporter protein 4
SCARF1	scavenger receptor class F member 1
SCN1B	sodium voltage-gated channel beta subunit 1
SELE	selectin E
SELL	selectin L
SELENOS	selenoprotein S
SEMA4D	semaphorin 4D
SERPINE1	serpin family E member 1
SGMS2	sphingomyelin synthase 2
SLAMF1	signaling lymphocytic activation molecule family member 1
SLC11A2	solute carrier family 11 member 2
SLC1A2	solute carrier family 1 member 2
SLC28A2	solute carrier family 28 member 2
SLC31A1	solute carrier family 31 member 1
SLC31A2	solute carrier family 31 member 2
SLC4A4	solute carrier family 4 member 4
SLC7A1	solute carrier family 7 member 1
SLC7A2	solute carrier family 7 member 2
SPHK1	sphingosine kinase 1
SRI	sorcin
STAB1	stabilin 1
TACR1	tachykinin receptor 1
TACR3	tachykinin receptor 3
TAPBP	TAP binding protein
TIMP1	TIMP metallopeptidase inhibitor 1
TLR1	toll like receptor 1
TLR2	toll like receptor 2
TLR3	toll like receptor 3
TNFAIP6	TNF alpha induced protein 6
TNFRSF1B	TNF receptor superfamily member 1B
TNFRSF9	TNF receptor superfamily member 9
TNFSF10	TNF superfamily member 10
TNFSF15	TNF superfamily member 15
TNFSF9	TNF superfamily member 9
TPBG	trophoblast glycoprotein
VIP	vasoactive intestinal peptide
